# *Ulopsina*, a Remarkable new Ulopine Leafhopper Genus from China

**DOI:** 10.1673/031.012.7001

**Published:** 2012-06-22

**Authors:** Wu Dai, Chandra A. Viraktamath, Yalin Zhang

**Affiliations:** ^1^ Key Laboratory of Plant Protection Resources and Pest Management, Ministry of Education, Entomological Museum, Northwest A&F University, Yangling, Shaanxi 712100, China; ^2^ Department of Entomology, University of Agricultural Sciences, GKVK, Bangalore 560065, India

**Keywords:** Auchenorrhyncha, distribution, morphology, new species, taxonomy

## Abstract

An unusual new cicadellid genus, *Ulopsina* gen. nov. and two new species, *U. sinica* sp. nov. and *U. szwedoi* sp. nov. from China are described, illustrated, and placed in the subfamily Ulopinae. The genus has characters of both the tribes Mesargini and Coloborrhinini, suggesting that the delimitation of these tribes may not be natural. The tribal placement of *Ulopsina* is uncertain. A checklist of the subfamily Ulopinae from China is also provided, and nine Chinese species designated under the genus *Moonia* are herein transferred to *Mesargus,* namely *Mesargus albomaculata* (Li) **comb. nov.**, *M. brevita* (Cai et Shen) **comb. nov.**, *M. castanea* (Kuoh) **comb. nov.**, *M. hei* (Cai et Shen) **comb. nov.**, *M. hirsuta* (Li) **comb. nov.**, *M. hyboma* (Cai et Kuoh) **comb. nov.**, *M. maculigena* (Kuoh) **comb. nov.**, *M. serrata* (Li and Zhang) **comb. nov.**, and *M. spinapenis* (Li and Zhang) **comb. nov.**

## Introduction

Leafhoppers constitute one of the largest families of insects, with more than 22,000 described species (Oman et al. 1990; Dietrich 2005). The subfamily Ulopinae, one of the 25 subfamilies of leafhoppers, consists of almost 40 genera and approximately 180 species (Szwedo and Gebicki 2001; Szwedo 2002), and is divided into the following five tribes: Ulopini, Mesargini, Cephalelini, Coloborrhinini, and Monteithini (Emeljanov 1996; Hamilton 1999). Ulopinae are apparently restricted to the Old World, where they are widely distributed in temperate and tropical regions. Although most of them are widespread in the Palearctic, Afrotropical, and Oriental regions, Cephalelini has a disjunct distribution in Australia and South Africa, and two species of Monteithiini are recorded from high elevations in New Guinea. Evans (1966) reviewed the Australian species, Linnavuori (1972) reviewed the Afrotropical species, Knight (1973) reviewed the New Zealand species, Emeljanov (1996) supplemented some and Szwedo (2002) reviewed the Palearctic species.

Examination of specimens under an ongoing project on Chinese leafhoppers revealed two new species of Ulopinae from Yunnan province that belong to a new genus with morphology intermediate between the tribes Mesargini and Coloborrhinini, recognized by Emeljanov (1996). This paper describes the new genus and the two new species, and discusses the relationship of the new genus with the different tribes of Ulopinae. Nine species designated under the genus *Moonia* are herein transferred to *Mesargus*, considering the fact that Vilbaste (1975) treated *Moonia* as a junior synonym of *Mesargus.* A checklist of Ulopinae, comprising three genera and 16 species from China, including the results of the present study, is provided.

## Materials and Methods

The type—specimens of the new species are deposited in the Entomological Museum of Northwest A&F University (NWAU), Institute of Zoology, Chinese Academy of Sciences, Beijing (IZCS), and Sun Yat-sen University (SYSU), as indicated under each species. Genitalia preparations were made by soaking the excised apex of the abdomen in cold 10% KOH for 8–10 hours. The apex of the abdomen was washed in distilled water and then transferred to glycerine for further dissection and examination. After examination, it was moved to fresh glycerine and stored in a micro vial pinned below the specimen.

All specimens were examined with a Leica ZOOM2000 stereomicroscope (www.leicamicrosystems.com). Drawings of male genitalia and external morphological characters were prepared using Nikon Eclipse 5Oi microscope (www.nikon.com) and a Nikon AFX-II stereomicroscope, respectively, both with a drawing tube attachment. Images were prepared using Automontage (version 5.02) with a QImaging Retiga 4000R High— Sensitivity IEEE 1394 FireWire Digital CCD Camera (QImaging, www.qimaging.com).

Morphological terminology follows Dietrich (2005), except for the leg chaetotaxy, which follows the system of Rakitov (1998). Absolute measurements, in millimeters (mm), are used for the body length taken from the apex of the head to the apex of folded forewings.

**Taxonomic accounts Checklist of the Ulopinae from China**
*Mesoparopia fruhstorferi* Matsumura, 1912 *Mesoparopia fruhstorferi* Matsumura, 1912: 28
Distribution: China, Vietnam.
*Mesoparopia nitobei* Matsumura, 1912 *Mesoparopia nitobei* Matsumura, 1912: 27
Distribution: China (Taiwan).
*Mesargus albomaculata* (Li) **comb. nov.**
*Moonia albomaculata* Li, 1989: 290
Distribution: China (Guizhou).
*Mesargus brevita* (Cai et Shen) **comb. nov.**
*Moonia brevita* Cai et Shen, 1999: 24
Distribution: China (Henan).
*Mesargus castanea* (Kuoh) **comb. nov.**
*Moonia castanea* Kuoh, 1986: 199
Distribution: China (Yunnan).
*Mesargus guttulinervis* (Kato)
*Moonia guttulinervis* Kato, 1933: 458
Distribution: China (Taiwan).
*Mesargus hei* (Cai et Shen) **comb. nov.**
*Moonia hei* Cai et Shen, 1998: 37
Distribution: China (Henan).
*Mesargus hirsuta* (Li) **comb. nov.**
*Moonia hirsuta* Li, 1989: 289
Distribution: China (Guizhou).
*Mesargus hyboma* (Cai et Kuoh) **comb. nov.**
*Moonia hyboma* Cai et Kuoh, in Lianget al, 1997: 324
Distribution: China (Hubei).
*Mesargus lata* (Kato)
*Moonia lata* Kato, 1933: 459
Distribution: China (Taiwan), Japan.
*Mesargus maculigena* (Kuoh) **comb. nov.**
*Moonia maculigena* Kuoh, 1986: 200
Distribution: China (Yunnan).
*Mesargus naevia* (Jacobi)
*Moonia naevia* Jacobi, 1944: 41
Distribution: China (Fujian).
*Mesargus serrata* (Li and Zhang) **comb. nov.**
*Moonia serrata* Li and Zhang, 2007 [in Liang et al. 2007:941]
Distribution: China (Hubei).
*Mesargus spinapenis* (Li and Zhang) **comb. nov.**
*Moonia spinapenis* Li and Zhang, 2007: 942
Distribution: China (Hubei).
*Ulopsina sinica* sp. nov.
Distribution: China (Guangxi, Yunnan).
*Ulopsina szwedoi* sp. nov.
Distribution: China (Yunnan).

The three genera known from China (including the genus described here) can be recognized by the following key.

**Key to genera of the Ulopinae from China**
1. Forewings convex, elytra—like; hind wings absent *Mesoparopia*

1. Forewings convex, elytra—like; hind wings absent *Mesoparopia*

**Figures 1–6.**
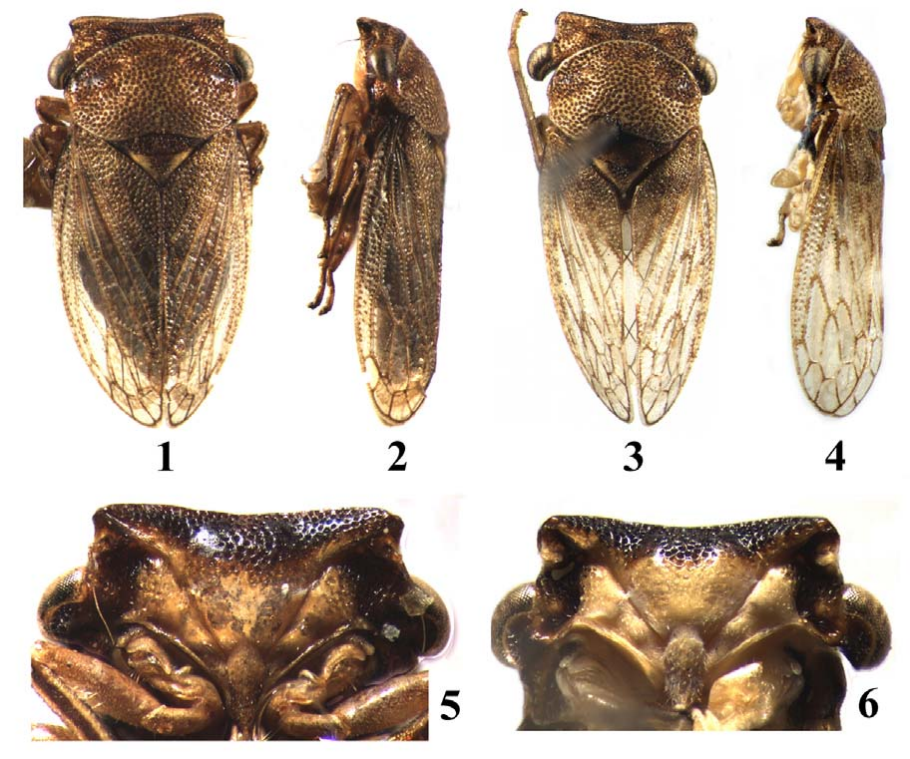
*Ulopsina* species. ( 1, 2, 5) *Ulopsina sinica* sp. nov.; (3, 4, 6) *Ulopsina szwedoi* sp. nov. (1,3) Habitus, dorsal view; (2,4) Habitus, lateral view; (5,6) Face. High quality figures are available online.

Forewings normal, not convex; hind wings fully developed 2
2. Hind wings with submarginal vein complete (Figure 22); head with crown relatively long (Figures 1, 3) *Ulopsina* gen. nov.
Hind wings with submarginal vein obsolete at apex; head with crown relatively short *Mesargus*

***Ulopsina* Dai, Viraktamath et Zhang, gen. nov.**
Type species: *Ulopsina sinica* Dai, Viraktamath et Zhang, sp. nov.
  Upper part of face, vertex, pronotum, and forewing strongly pitted. Head with transocular width wider than pronotum; crown in lateral view declivous, anterior margin broadly produced in front of eyes, and concave; lateral margins in front of eyes concave, exposing pedicel of antennae. Eyes projecting. Ocelli on vertex nearer to median line of head than to adjacent eye, closer to anterior margin of head than to posterior and not surrounded by carinae or ridges; callosities present behind each ocellus. Face sparsely pubescent. Frontoclypeus V—shaped, much wider near upper margin of face, not extending onto crown. Antennal ledges very prominent; antennae hanging down from roof—like area adjacent to eye. Genae narrow, not reaching apex of clypellus. Upper margins of genal sulcus rather vertical, extending to mesal margin of antennal pit. Crescent—shaped callosity on either side of median line on upper part of face. Clypeal sulcus arcuate. Clypellus projecting well beyond genal curve, apically narrowed, conically rounded. Labium reaching mesocoxae. Pronotum with anterior half declivous, uniformly coarsely pitted, with anterior margin arcuate and posterior margin concave; lateral margins carinate, a callosity on either side of median line in anterior half. Scutellum 0.5–0.75 times as long as pronotum. Anepi sterna and katepi sterna smooth, without typical carina or conical outgrowth. Forewings with clavus coriaceous, apical two—thirds of corium transparent with prominent raised venation; each vein margined by pits; appendix absent; vein R bifurcate at midlength of forewings, M bifurcate after branching of R; with five apical and three subapical cells, claval veins separate. Hind wings with four distal apical cells, with well developed r—m and m—cu cross veins. Fore femora AV row with two prominent setae; intercalary row irregular. Hind femora with 2+0 macrosetae at distal end in addition to small setae. Hind tibiae with 6 AD and 4 PD stout setae in addition to number of hairlike setae on PD becoming more numerous distally; stout PD setae arising from distal half of hind tibiae, AV and PV with many fine setae. Hind basitarsi conically rounded caudally without distal transverse row of setae; surface with irregularly arranged hair—like setae.
  Male pygofer with long, well—sclerotized dorsal surface; valve fused with terga; caudal lobe with sclerotized process rugose; mesal circular area with five short, stout setae. Anal tube short, without anal collar or process. Subgenital plates not segmented nor with basal suture, without macrosetae. Styles elongate, anterior part shorter than posterior part, gradually tapered caudally with distal end dorsally upturned, with series of setae on lateral margin. Connective plate—like, anterior margin wider than posterior margin. Aedeagus with well—developed dorsal apodeme, shaft cylindrical with pair of subapical asymmetrical processes and basal unpaired lateral process; gonopore apical.
**Remarks.** *Ulopsina* gen. nov. has forewing venation usual for Ulopinae and lacks accessory cross veins and hence may be placed in the tribe Mesargini (Emeljanov 1996). However, the hind wing venation has features of the Coloborrhinini and Cephalelini, in that the apical cells are closed. In Coloborrhinini, the forewing has reticulate venation, and the subgenital plates are neither divided nor have basal suture. The pygofer of *Ulopsina* gen. nov. is as in Ulopini and Cephalelini, but the aedeagus is similar to that found in Mesargini, differing only in the presence of subapical processes and the basal long process on the shaft. The genital styles are similar to those found in Coloborrhinini, with tapering apex not roundly widened as in Mesargini. External features, such as the comparatively longer head with ocelli placed on the vertex and the carinate anterior margin resemble those of Coloborrhinini. The genus also has some resemblance to *Radhades* Distant, but differs in having a more flattened face and more prominently carinate anterior head margin compared to the latter. *Ulopsina* gen. nov. differs from *Mesoparopia* in having well-developed hind wings. Because of these peculiar features, the new genus cannot be assigned to any of the tribes so far recognized in the subfamily.
**Etymology.** The name of the genus, which is feminine, was derived by combining *Ulop-*, a common suffix for genus names in this subfamily, with *-sino*, meaning Chinese.

**Key to species of *Ulopsina* Dai, Viraktamath et Zhang, gen. nov.**
1. Pygofer with caudal lobe quadrate (Figure 7); aedeagal shaft with basal process not sinuous in lateral view, with apical spine—like extension dorsally (Figure 11) *U- sinica* sp. nov.
Pygofer with caudal lobe not quadrate, but ventral aspect convexly rounded (Figure 14); aedeagal shaft with basal process sinuous in lateral view lacking spine-like dorsal extension (Figure 18) *U*-*. szwedoi* sp. nov.

***Ulopsina sinica* Dai, Viraktamath et Zhang, sp. nov. (Figures 1, 2, 5, 7–13, 21–27)**
**Material examined.** Holotype: Male, China, Guangxi, Guiping, Xishan, 18 May 1959, Guangxi team (SYSU). Paratype: 1 male, China, Yunnan, Rezhisuo, 11 April 1982 (NWAU).
**Measurements.** Male 5.8–6.0 mm long, 2.0– 2.5 mm wide across anterior margin of head; 3.0 mm wide across eyes.
**Coloration.** Pale brown. Anterior margin of head narrowly dark fuscous, submargin ochraceous; T—shaped marking with short stem and callosities dark—brown; transverse spot adjacent to each eye on vertex, ochraceous. Upper part of face including antennal cavities chocolate—brown, the rest ochraceous. Anterior area of pronotum with a few irregular dark—brown areas including depressed areas and callosities. Scutellum darker than pronotum with marginal spot on either side of median impressed line pale ochraceous. Forewings with claval commissure, one submarginal streak along costal margin, and apical veins, dark brown. All tibiae dark brown with edges ochraceous; tarsi brown.
**Male genitalia.** Pygofer slightly depressed, dorsal margin declivous in posterior half, ventral margin rounded, caudal lobe quadrate, darkly pigmented with rugose surface; short pygofer apodeme of uniform length on anterior margin along dorsal and lateral ends, extending slightly posteriorly along median line on dorsum. Valve large. Subgenital plates almost parallel—sided with rounded apex. Anal segments 10 and 11 well—sclerotized and pigmented. Styles linear, caudal part longer than anterior part, in lateral view tapering caudally to pointed tip, upcurved distally. Aedeagus with dorsal apodeme as long as basal process of shaft; shaft with hyaline ridge along entire length to subapical process on right side; basal process pigmented, straight, asymmetrically arising on ventral side on left side; apex with broad crenulate flat plate with caudal spine-like extension; paired subapical processes with broad transparent base, curved laterally and then anteriorly with anterior margin serrate; gonopore apical, gonoduct prominent.
**Remarks.** The paratype male has most of the veins marked with brown interrupted by white. This is a more robust and darker species compared to *U. szwedoi* sp. nov (see below).
**Etymology.** The name of this species is derived from Sinica meaning Chinese.

***Ulopsina szwedoi* Dai, Viraktamath et Zhang, sp. nov. (Figures 3, 4, 6, 14–20)**
**Material examined**. Holotype: male, Yunnan, Xishuangbanna, 620–650 m, 2 May 1959, Zhang Facai (IZCS); Paratype: 1 male, Yunnan, Xishuangbanna, 1200–1400 m, 25 May 1958, Meng Xuwu (IZCS).
**Measurements.** Male 5.5 mm long, 2.0 mm wide across anterior margin of head, 2.7 mm wide across eyes.
**Coloration.** Similar to *U. sinica* but paler, lacking brown markings on forewings and legs.
**Male genitalia.** Similar to *U. sinica* with following differences. Caudal lobe of pygofer rounded in lateral view, but more transverse and bilobed. Basal process of aedeagal shaft longer and sinuous lacking spine—like extension of the expanded area; paired subapical process directed dorsally and shorter.
**Remarks.** Externally, *U. szwedo* is very similar to *U. sinica*, but smaller and paler.
**Etymology.** The new species is named after Dr. Jacek Szwedo, Polish Academy of Sciences, Warszawa, Poland, in recognition of his contribution to the Auchenorrhyncha.

**Figures 7–1:**
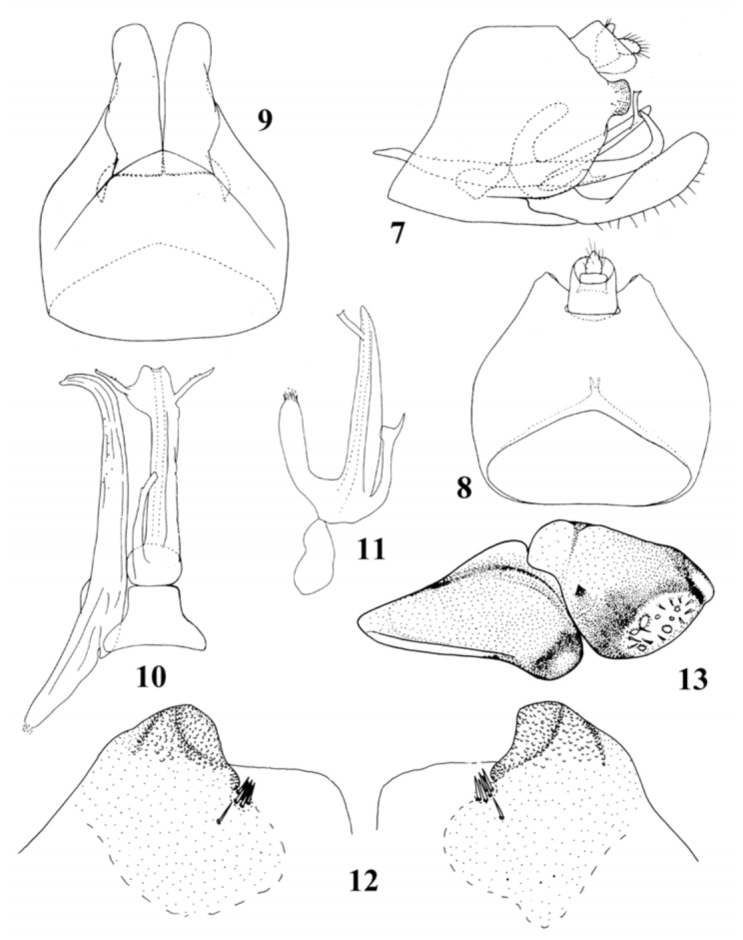
3. *Ulopsina sinica* sp. nov. (7) Pygofer, lateral view; (8) Pygofer, dorsal view; (9) Pygofer, ventral view; (10) Aedeagus, connective and left style, ventral view; (11) Aedeagus, lateral view; (12) Magnified view of caudal lobe of Pygofer, dorso—caudal view; (13) Anepisternum and katepisternum, lateral view. High quality figures are available online.

**Figures 14–20.**
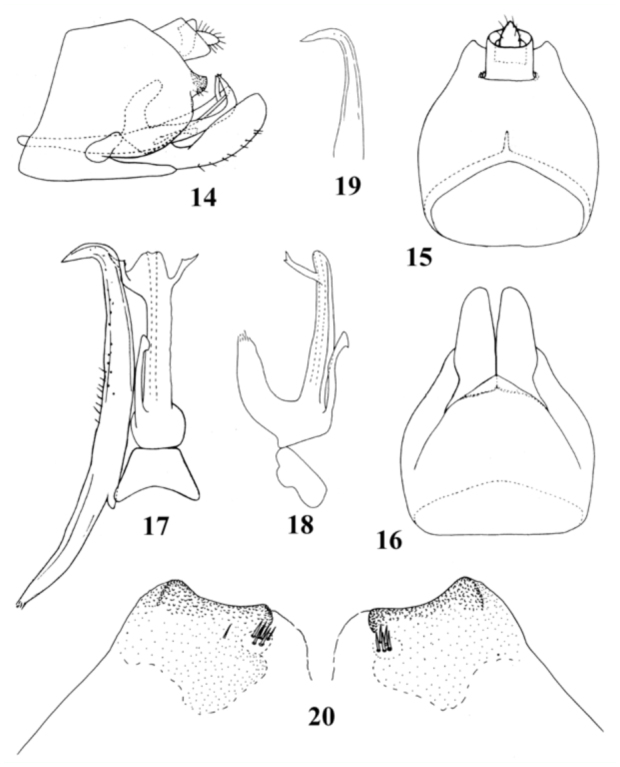
*Ulopsina szwedoi* sp. nov. (14) Pygofer, lateral view; (15) Pygofer, dorsal view; (16) Pygofer, ventral view; (17) Aedeagus, connective and left style, ventral view; (18) Aedeagus, lateral view; (19) Apex of style, lateral view; (20) Magnified view of caudal lobe of pygofer, dorso—caudal view. High quality figures are available online.

**Figures 21–27.**
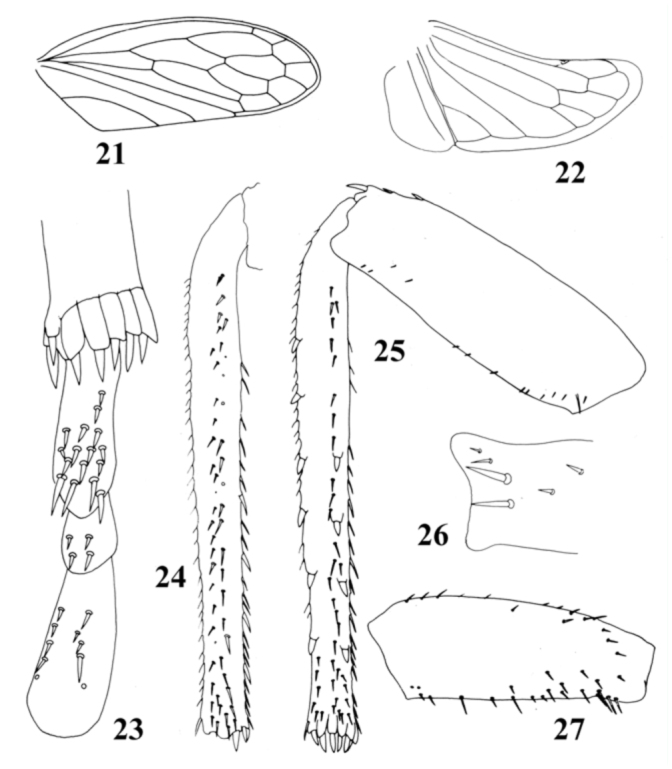
*Ulopsina sinica* sp. nov. (21) Forewing; (22) Hindwing; (23) Apex of hind tibia and hind tarsomeres, ventral view; (24) Hind tibia, posterior surface; (25) Hind femur and tibia, dorsal surface; (26) Apex of hind femur, dorsal view; (27) Fore femur, anterior surface. High quality figures are available online.
